# Second Cancers in a Patient with Gastric MALT Lymphoma

**DOI:** 10.1155/2020/1213596

**Published:** 2020-05-13

**Authors:** Lucy Navsaria, Alfonso Badillo, Michael Wang

**Affiliations:** Department of Lymphoma and Myeloma, Division of Cancer Medicine, The University of Texas MD Anderson Cancer Center, Houston, TX, USA

## Abstract

Mucosa-associated lymphoid tissue (MALT) lymphoma is an extranodal low-grade B-cell lymphoma, which is thought to arise from a background of chronic immune stimulation, bacterial, viral, or autoimmune stimuli. Treatment advances have increased the number of MALT lymphoma survivors, but there is still debate as to whether these patients are at a higher risk of developing second cancers. This is a case of a long-surviving (>20 years) patient with multiple diagnosed malignancies following MALT lymphoma. We describe how modern oncological treatment plans can provide patients with prolonged survival and increased quality of life despite increasing age and multiple malignancies.

## 1. Introduction

Marginal zone lymphomas (MZL) [1], according to the WHO classification, are divided into three main entities: extranodal marginal zone B-cell lymphoma of the mucosa-associated lymphoid tissue (MALT lymphoma), nodal MZL, and splenic MZL. MALT lymphomas are generally low-grade B-cell malignancies with an indolent clinical course. Ninety percent of these are associated with the bacteria *Helicobacter pylori* [2]. MALT lymphoma is thought to arise from postgerminal center memory B cells, which have the capacity to differentiate into marginal zone and plasma cells [3]. There has been increasing evidence to suggest that many cases of MALT lymphoma arise from a background of chronic immune stimulation, bacterial, viral, or autoimmune stimuli. These tumor cells may express CD20(+), CD5(−), CD10(−), BCL2(+), CD23(−), or cyclin D1(−) [4].

MALT lymphomas account for 8% of all newly diagnosed lymphomas [1]. The gastrointestinal tract (GIT) is the site most commonly affected by MALT lymphomas. Although they most commonly occur in the stomach, they can develop in almost any mucosal site [5]. MALT lymphoma has the highest incidence of occurrence in people between 50 and 60 years old [6].

Modern oncological treatments have increased the number of MALT lymphoma survivors over the last few decades; however, researchers debate whether patients with gastric MALT lymphoma are at a higher risk of developing secondary malignancies. Zucca and colleagues [7] suggested that a cancer-prone phenotype may exist in patients with MALT lymphoma, as they observed a high incidence (20%) of other neoplasms in patients who had been diagnosed with low-grade gastric MALT lymphoma. In 2014, Tajika and colleagues [8] followed 146 patients with gastric MALT lymphoma over 74 months and found that 27 tumors were detected in 22 patients. Nine of those were detected concomitantly with diagnosis of gastric MALT lymphoma, and 18 tumors were detected following the initial diagnosis. Four patients in the study had two secondary malignancies each. Overall, the group experienced an increased risk of developing second malignancies during the follow-up period (SIR 2.26 (1.21–3.30)), supporting the conclusion that patients diagnosed with gastric MALT lymphoma have an increased risk for second malignancies [8].

Here, we report on the case of a long-surviving (>20 years) patient with multiple diagnosed malignancies following MALT lymphoma, and we describe how modern oncological treatment plans can provide patients with prolonged survival and increased quality of life despite increasing age and multiple malignancies.

## 2. Case Report

Our patient first presented to MD Anderson Cancer Center (MDACC) in 1994 and was first diagnosed with *H. pylori*-negative gastric MALT lymphoma, via biopsy PCR, when he was found to have a large 5 × 5 multiloculated ulcerative mass in his stomach. He achieved complete remission following a subtotal gastrectomy with a Billroth II gastrojejunostomy, performed at MDACC, and six cycles of CHOP (cyclophosphamide, doxorubicin, vincristine, and prednisone) chemotherapy. He relapsed in 1998 and achieved complete remission following radiation therapy (total dose of 39.6 Gy, given in 22 fractions).

**Figure 1:**
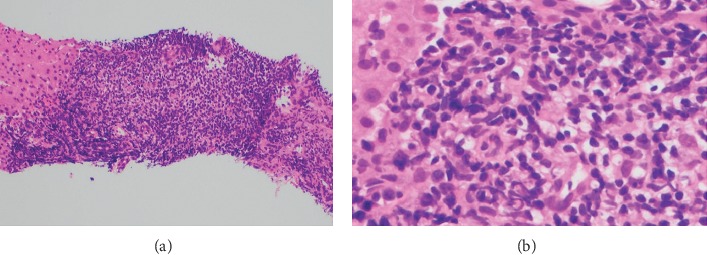
Low-grade marginal zone lymphoma confirmed by biopsy of liver (CD5− and CD10−).

In May 2013, the patient began experiencing right-sided epigastric pain, generalized abdominal cramping, and nausea. An upper endoscopy was performed, and he was found to have gastric cancer T2N0. Following the endoscopy, he underwent a CT scan of his chest, abdomen, and pelvis, which revealed extensive emphysematous changes in both lungs with a calcified granuloma and a spiculated mass in the left lower lobe. The chest, abdomen, and pelvis CT also revealed a thickened loop of small bowel in the right upper quadrant directly inferior to the gallbladder with ileocolic mesenteric adenopathy. Interventional radiology confirmed the spiculated mass in the lung to be squamous cell carcinoma (SCC) of the lung. A biopsy of the mesenteric nodes and liver confirmed the presence of CD5− and CD10− low-grade marginal zone lymphoma (Figure 1).

In August 2013, to treat the lung cancer, the patient elected to participate in an SBRT (stereotactic body radiotherapy) vs. SBPT (stereotactic body proton therapy) clinical trial protocol, and he was randomized to the SBPT arm. His left lung was treated with a total dose of 50 Gy in four fractions using a 4-beam proton arrangement, prescribed to 96%. His B-cell low-grade marginal zone lymphoma was addressed in September 2013 with two cycles of rituximab and bendamustine in hopes of entering remission prior to surgery for the gastric cancer diagnosed in May 2013. In September 2013, he underwent a total gastrectomy for the gastric cancer (T2N0), followed by five months of adjuvant chemotherapy with oxaliplatin and 5-FU (fluorouracil).

In December 2014, a PET/CT scan of his chest, abdomen, and pelvis showed no recurrent lung cancer, no recurrent lymphoma, no gastric cancer, and no remote metastasis.

To the best of our knowledge, this is the first reported case of recurrent MALT lymphoma with two synchronous second primary unrelated cancers.

## 3. Discussion

MALT lymphoma is a low-grade lymphoma originating from the mucosa-associated lymphoid tissue and is typically an indolent NHL strongly associated with *H. pylori* [1]. MALT lymphoma is the most common subtype of marginal zone lymphoma, accounting for 70% of all MZLs [5]. MALT lymphoma can originate at any extranodal site and arises in organs that normally lack lymphoid tissue (stomach, intestine, thyroid, lung, and skin). MALT lymphomas usually maintain an indolent course with 10-year recurrence-free rates of 76% and survival rates of 87% [9]. Little, however, is known about the rate of second malignancies following complete remission of MALT lymphoma. Tajika and colleagues [8] concluded that, compared to the general population, patients who had gastric MALT lymphoma were at an increased risk for developing second malignancies, including solid and hematological tumors. Furthermore, Tajika and colleagues [8] found that most patients with gastric MALT lymphoma who experienced additional malignancies did not receive additional modern treatment regiments nor appropriate oncological follow-up. This, however, was not the case with our patient. We reported a case in which a patient with MALT lymphoma stage IIa with multiple recurrences despite various modern treatment plans and follow-up eventually achieved complete remission after two secondary cancers and two recurrences of lymphoma following the initial diagnosis of MALT lymphoma in 1994.

Today, successful modern oncology treatment plans have improved the life expectancy of those affected by secondary malignancies despite increasing age. Radiotherapy has been shown to be a promising therapy for residual or recurrent gastric MALT lymphoma. In a 10-year follow-up study, Ohkuba and colleagues found that patients with gastric MALT lymphoma who were treated with radiotherapy experienced good long-term control of their disease [10]. Radiotherapy thus may be the favored choice for patients with *H. pylori*-negative gastric MALT lymphoma, like our patient, or those who experience relapse after treatment. The scientific consensus is that excellent disease control can be achieved with the use of moderate dose 24–30 Gy given over three to four weeks. This moderate radiation dose is associated with only mild/reversible toxicities [9], allowing it to be a viable option for patients with gastric MALT lymphoma.

Today, the first-line treatment for *H. pylori*-negative gastric MALT lymphoma is antibiotic therapy [11]. Various studies have now reported that certain patients respond to antibiotic therapy even with *H. pylori*-negative MALT lymphoma. Raderer et al. [12] reported that five out of six *H. pylori*-negative gastric MALT lymphoma patients responded to antibiotic therapy. Contrary to the recent reports of positive response to antibiotic treatment with *H. pylori*-negative gastric MALT lymphoma, reports conducted in the late 1990s and early 2000s stated that *H. pylori*-negative gastric lymphoma did not respond to antibiotic therapy [13, 14]. Given our patient was first diagnosed with *H. pylori*-negative MALT lymphoma prior to the published reports of response with antibiotic is likely the reason for why antibiotic therapy was not pursued as a treatment modality.

A study by Montalban and colleagues [15] suggested that patients with gastric MALT lymphoma may have other malignancies. Both chemotherapy and radiotherapy for the initial gastric MALT lymphoma are likely to play a role in the development of second cancers following this diagnosis. Some studies suggest that age plays a role in the development of second cancers [16]; in a meta-analysis of all second primary malignancies in NHLs [17], a younger age at diagnosis was associated with development of secondary solid tumors. Additionally, genetic factors and lifestyle factors may also influence development of second cancers. Travis and colleagues [18] observed that a history of smoking, an important environmental risk factor, was associated with a greater risk of lung cancer following treatment for lymphoma.

Numerous papers describe the synchronous occurrence of gastric MALT lymphoma and gastric adenocarcinoma [19, 20], mainly as a result of the pathogenic role of *H. pylori* in both conditions. Although our patient in this case was found to be *H. pylori*-negative at initial diagnosis, *H. pylori* is likely to be the most important factor in the development of gastric cancer as a second cancer following gastric MALT lymphoma. The oncologic pathogenesis with *H. pylori* is different in gastric MALT lymphoma and gastric carcinoma—*H. pylori* in MALT lymphoma is a multistage process starting with the infection of *H. pylori*, which results in the recruitment of B cells, T cells, and other inflammatory cells to the gastric mucosa, and the inflammatory cells are thus stimulated by the *H. pylori*-specific T cells and undergo malignant transformation [21]. In gastric carcinoma, however, *H. pylori* induces an atrophic gastritis, dysplasia, and metaplasia with gastric cancer as a final step. *H. pylori* being an important underlying component in both MALT lymphoma and gastric carcinoma is a plausible reason for the predisposition to gastric carcinoma following MALT lymphoma.

## 4. Conclusion

The fact that relapse can occur more than 20 years following complete remission highlights the importance of following current guidelines [22], which suggest follow-up strategies to align with those used for other indolent B-cell malignancies, including physical examinations, performance status assessments, hematological examinations, and endoscopic follow-ups. Histological evaluations with repeat biopsies are an essential follow-up method to ensure there is no possibility of persistent disease or any early epithelial changes, which could indicate cancerous activity [23]. Although treatment options for MALT lymphoma have improved, given the heterogeneous nature of the malignancy, clinical trials need to be conducted to identify and establish a standardized treatment plan for those who experience relapsed gastric MALT lymphoma. Nevertheless, we believe that multidisciplinary team care and current advancements have enabled our patient to achieve complete remission for each subsequent malignancy and remain disease-free despite his age and multiple malignancies.
